# Hinokitiol, a Natural Tropolone Derivative, Offers Neuroprotection from Thromboembolic Stroke *In Vivo*

**DOI:** 10.1155/2013/840487

**Published:** 2013-10-27

**Authors:** Thanasekaran Jayakumar, Wen-Hsien Hsu, Ting-Lin Yen, Jun-Yun Luo, Yu-Cheng Kuo, Tsorng-Harn Fong, Joen-Rong Sheu

**Affiliations:** ^1^Department of Pharmacology, Graduate Institute of Medical Sciences, Taipei Medical University, 250 Wu-Hsing Street, Taipei 110, Taiwan; ^2^Department of Surgery, Wan-Fang Hospital, Taipei Medical University, 111 Hsing-Long Road, Taipei 110, Taiwan; ^3^Department of Anatomy, College of Medicine, Taipei Medical University, Taipei 110, Taiwan

## Abstract

Hinokitiol (**β**-thujaplicin), a tropolone-related compound found in the heartwood cupressaceous plants, is widely used in hair tonics, tooth pastes, cosmetics, and food as an antimicrobial agent. Increasing evidence has confirmed that hinokitiol exhibits anticancer activity in a variety of cancers through inhibition of cell proliferation. In the present study, we have investigated the neuroprotective effect and mechanisms of hinokitiol in rats against middle cerebral artery occlusion (MCAO)-induced thromboembolic stroke. Treatment with hinokitiol (0.2 and 0.5 mg/kg; intraperitoneally) 30 min before MCAO dose dependently attenuated cerebral ischemia and improved neurobehavioral deficits in cerebral ischemic rats. Intraperitoneal administration of hinokitiol significantly reduced infarct size compared to that in control rats. MCAO-induced focal cerebral ischemia was associated with increased expressions of hypoxia-inducible factor (HIF)-1**α**, inducible nitric oxide synthase (iNOS), tumor necrosis factor (TNF)-**α**, and active caspase-3 in ischemic regions. However, these expressions were obviously inhibited by hinokitiol (0.2 and 0.5 mg/kg) treatment. This study demonstrates for the first time that in addition to being originally considered as an agent against microbes and variety of cancers, hinokitiol possesses potent neuroprotective activity. This activity is mediated, at least in part, by inhibition of inflammatory responses (i.e., HIF-1**α**, iNOS expression) and apoptosis (i.e., TNF-**α**, active caspase-3), resulting in a reduction of infarct volume and improvement in neurobehavior in rats with cerebral ischemia. Therefore, the therapeutic potential of hinokitiol may lead to novel role for treatment or prevention of ischemia/reperfusion injury-related disorders.

## 1. Introduction

Stroke is the third leading cause of death, ranking after heart disease and cancer and the primary cause of adult disability worldwide [1, 2]. It is also a major health concern in the industrialized countries. In spite of major advances of neuroprotective therapeutic approaches for treating ischemic stroke over the last decade, stroke is still a serious problem for which effective drug therapy is not yet available [3]. The recombinant tissue plasminogen activator (rt-PA), a thrombolytic agent, has long been used to improve the outcomes of acute ischemic stroke patients by restoring cerebral blood flow (CBF) [4]. Nevertheless, its use remains limited to less than 5% patients due to its narrow therapeutic window [5]. Furthermore, clinical data showed that whatever its administration time, rt-PA increases the risk of hemorrhagic transformation (HT) [6]. Clinical practice also showed that rt-PA does not induce recanalization in all ischemic patients. Therefore, research has been directed at finding alternative therapies that target neurons downstream of the site of the thrombosis in an effort to salvage them from the vicious circle of inflammation, necrosis, and apoptosis, all of which are implicated in ischemic stroke [7]. Natural products are a prolific source of bioactive agents of different structure and varying biological activity. In the search for neuroprotective agents from natural sources, a number of plant extracts and several natural products isolated from them have been reported to provide neuroprotection against ischemic stroke [8].

Hinokitiol, also known as *β*-thujaplicin, is a tropolone derivative found in the heartwood of cupressaceous plants [9]. As an iron-chelating compound, it triggers apoptosis via activation of caspase-3 [10] and exerts a spectrum of biological effects including differentiation-inducing [11], anti-inflammatory [12], antibacterial [13], antifungal [14], and antioxidant [15] capacities, as well as antitumor activity [16]. Hinokitiol has also been widely used in hair tonics, tooth pastes, cosmetics, and food as an antimicrobial agent [17]. Hinokitiol has been shown to suppress tumor growth by inhibiting cell proliferation and inducing apoptosis in various carcinoma cell lines [16]. Hinokitiol regulates immune cell function by inhibiting the production of TNF-*α* from LPS-stimulated macrophages via inhibition of NF-*κ*B activity [9]. Our recent study also clearly demonstrates that hinokitiol possesses antiplatelet activity by inhibiting the PLCg2 and/or PKC cascades, and hydroxyl radical formation, followed by suppressing the activation of MAPKs and Akt [18]. Despite such wide range of roles in signaling pathways, there is no report about the direct evidence of the neuroprotective effect of hinokitiol. The purpose of this contribution is thus to demonstrate whether hinokitiol has a neuroprotective effect against thromboembolic stroke in rats. 

## 2. Materials and Methods

### 2.1. Materials

**Figure 1:**
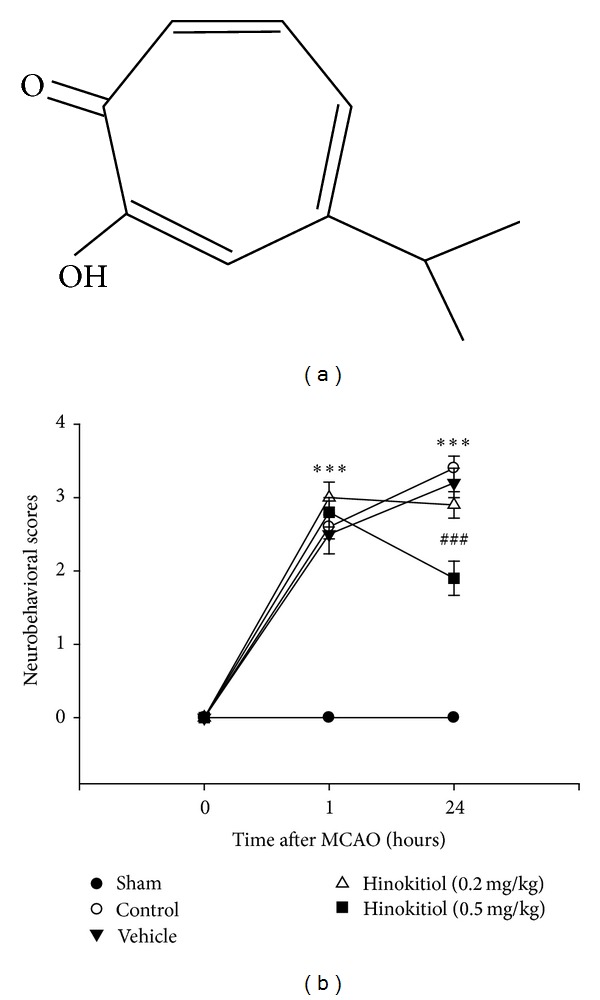
(a) Chemical structure of hinokitiol (C_10_H_12_O_2_, MW: 164.2). (b) Effects of hinokitiol on the MCAO-induced neurobehavioral deficits scores. Neurobehavioral scores were recorded at 1 and 24 h after MCAO. Data are expressed as the means ± SEM (*n* = 10). ***P < 0.001 compared to sham-operated group; ^###^ *P* < 0.001 compared to solvent-treated group.

Hinokitiol (Figure 1(a), 99%), collagen (type I), cremophor EL, 5,5-dimethyl-1 pyrroline N-oxide (DMPO), and bovine serum albumin (BSA) were purchased from Sigma (St. Louis, MO). Hinokitiol was dissolved in a solvent (cremophor : ethanol : DMSO at 1 : 1 : 4) for the present study.

### 2.2. Animals

Male Wistar rats (250~300 g) were used in this study. All animal experiments and care were performed according to the *National Research Council Guide for the Care and Use of Laboratory Animals* and were approved by the Institutional Animal Care and Use Committee (IACUC) of Taipei Medical University (number LAC-98-0088). Before undergoing the experimental procedures, all animals were clinically normal, free of apparent infection or inflammation, and showed no neurological deficits.

### 2.3. Experimental Groups

Animals were divided into 5 groups: (1) a sham-operated group; (2) a group orally treated with an isovolumetric solvent (distilled water) for 14 days, followed by thromboembolic occlusion; (3) a group orally treated with solvent (cremophor : ethanol : DMSO at 1 : 1 : 4) for 14 days, followed by thromboembolic occlusion; 4 and 5 groups treated with a single dose of hinokitiol (0.2 and 0.4 mg/kg, resp.), followed by thromboembolic occlusion. Rats received the isovolumetric normal saline, solvent, or hinokitiol (0.2 or 0.5 mg/kg) 30 min before MCAO was performed.

### 2.4. Thromboembolic Occlusion of the Middle Cerebral Artery (MCA) in Rats

Animals were anesthetized with a mixture of 75% air and 25% O_2_ gases containing 3% isoflurane. The rectal temperature was maintained at 37 ± 0.5°C. The right MCA was occluded with a blood clot as an embolus. The method of embolus preparation and surgical procedures were slightly modified from a previous description by Krueger and Busch [19]. Briefly, arterial blood (0.6 mL) was withdrawn from a femoral catheter in a 1 mL syringe. The blood was immediately injected into PE-50 tubes. The tubes were kept at 4°C for approximately 22 h, and the thread-like clots were removed and placed in a phosphate-buffered saline (PBS)-filled dish. The clots were then washed to remove blood cells. Washed portions of the clots were transferred to fresh dishes, and the washing process was repeated until the PBS remained clear. These clot sections were cut into 30 mm-long fragments and then drawn up with the PBS solution into a PE-50 catheter.

The common carotid artery (CCA) was identified, and approximately 1 cm of the external carotid artery (ECA) was ligated and cut. Subsequently, the pterygopalatine artery (PA) was clamped with a 10 mm microaneurysm clamp, and the CCA was similarly clamped before the carotid bifurcation. The internal carotid artery (ICA) was then clamped between the carotid bifurcation and the PA. Next, the PE-50 catheter containing the clot was introduced approximately 5 mm into the previously cut ECA and tied in place with sutures. The ICA clamp was removed, and the clot was flushed into the ICA over a period of approximately 5 s. The PA clamp was removed, and the rat was left in this condition for 1 h.

At the end of this period, the catheter was removed from the ECA stump, an unperturbed portion of the ECA close to the bifurcation was tied off, and the incision was closed. After closure of the operative sites, the animals were allowed to wake from the anesthesia. An observer blinded to the identity of the groups assessed the neurological deficits at 1 and 24 h after reperfusion (before being euthanized) by forelimb akinesia (also called the postural tail-hang) test, whereas a spontaneous rotational test was used as a criterion for evaluating the ischemic insult [20]. Animals not showing any behavioral deficits at the above time points after reperfusion were excluded from the study. On the other hand, reperfusion was also ensured by an improvement in ipsilateral local blood flow to at least 60% of the baseline following an initial sharp decrease to about 50%~60% of the baseline caused by MCA occlusion as determined using a continuous laser Doppler flow meter (LDF; Oxford Array, Oxford Optronix, Oxford, UK) with a standard needle probe (pp-051).

Rats were euthanized by decapitation after 24 h of reperfusion. The brains were cut into 2 mm coronal slices starting 1 mm from the frontal pole. Each stained brain (2% 2,3,5-triphenyltetrazolium; TTC) slice was drawn using a computerized image analyzer (Image-Pro plus). The calculated infarct areas were then compiled to obtain the infarct volume (mm^3^) per brain. Infarct volumes were expressed as a percentage of the contralateral hemisphere volume using the formula (the area of the intact contralateral [left] hemisphere − the area of the intact region of the ipsilateral [right] hemisphere) to compensate for edema formation in the ipsilateral hemisphere [21].

### 2.5. Expressions of HIF-1*α*, iNOS, TNF-*α*, and Active Caspase-3 in Thromboembolic Occlusion-Insulted Brain

Expressions of HIF-1*α*, iNOS, TNF-*α*, and active caspase-3 in the ischemic brain at 24 h after thromboembolic occlusion-reperfusion injury were analyzed by immunoblotting as described by Rodrigo et al. [22], with minor modifications. Thromboembolic occlusion-insulted and sham-operated rats were anesthetized with chloral hydrate (400 mg/kg, i.p.), and then the apex of the heart was penetrated with a profusion cannula inserted through the left ventricle into the ascending aorta. Perfusion with ice-cold PBS was performed, and an incision was made in the right atrium for venous drainage. Brains were freshly removed and sectioned coronally into four sequential parts from the frontal lobe to the occipital lobe. The third of four parts of the right hemisphere was separately collected, snap-frozen in liquid nitrogen, and stored at −70°C. The frozen tissues were placed in homogenate buffer and homogenized and then sonicated for 10 s three times at 4°C. The sonicates were subjected to centrifugation (10,000 ×g).

The supernatant (50 *μ*g protein) was subjected to sodium dodecylsulfate polyacrylamide gel electrophoresis (SDS-PAGE) and electrophoretically transferred to polyvinylidene difluoride (PVDF) membranes (0.45 *μ*m, Hybond-P, Amersham). After incubation in blocking buffer and being washed three times with TBST buffer (10 mM Tris-base, 100 mM NaCl, and 0.1% Tween 20; pH 7.5), blots were treated with an anti-HIF-1*α* polyclonal antibody (pAb, 1 : 1000; R&D, Minneapolis, MN), an anti-iNOS monoclonal antibody (mAb; 1 : 3000, BD Biosciences, San Jose, CA), an anti-TNF-*α* pAb (1 : 1000; Cell Signaling, Beverly, MA), and an antiactive caspase-3 pAb (1 : 250; Biovision, Mountain View, CA) or an anti-*α*-Tubulin mAb (1 : 2000; Santa Cruz Biotechnology, Santa Cruz, CA) in TBST buffer overnight. Blots were subsequently washed with TBST and incubated with a secondary horseradish peroxidase (HRP)-conjugated goat anti-mouse mAb ordonkey anti-rabbit immunoglobulin G (IgG) (Amersham) for 1 h. Blots were then washed, and the immunoreactive protein was detected using film exposed to enhanced chemiluminescence (ECL) detection reagents (ECL^+^ system; Amersham). The bar graph depicts the ratios of semiquantitative results obtained by scanning reactive bands and quantifying the optical density using videodensitometry (Bio-1D vers. 99 image software).

### 2.6. Statistical Analysis

Experimental results are expressed as the mean ± SEM and are accompanied by the number of observations. The experiments were assessed by the method of analysis of variance (ANOVA). If this analysis indicated significant differences among the group means, then each group was compared using the Newman-Keuls method. A *P* value of <0.05 was considered statistically significant.

## 3. Results

### 3.1. Effects of Hinokitiol on Neurological Deficit Score and Ischemic Brain Damage

Following stroke, animals subsequently exhibit a variety of neurological deficits. It is very significant to evaluate neurological function outcome after stroke. The Bederson scale is a global neurological assessment that was developed to measure neurological impairments following stroke [23]. Our results revealed that hinokitiol could improve neurological behavior disturbance based on neurological deficit scores.

The neurological deficit of vehicle-treated, hinokitiol-treated, and sham-operated rats, evaluated 24 h after MCAO, are shown in Figure 1(b). The neurological scores were significantly increased (*P* < 0.001) after 24 hr of ischemia as compared to sham-operated rats and after 1 hr of ischemia. Treatment of hinokitiol (0.2 and 0.5 mg/kg) significantly improved the neurological deficit in MCAO-induced rats when compared to vehicle-treated rats. Moreover, the neurological scores were significantly (*P* < 0.001) effected by hinokitiol treatment at a dose of 0.5 mg/kg than that of the solvent and 0.2 mg/kg hinokitiol-treated groups (Figure 1(b)). No score was found in the sham-operated rats or in the hemisphere contralateral to the ischemic side.

### 3.2. Effects of Hinokitiol on MCAO-Induced Focal Cerebral Infarction Volume in Embolic Occlusion-Induced Rats

**Figure 2:**
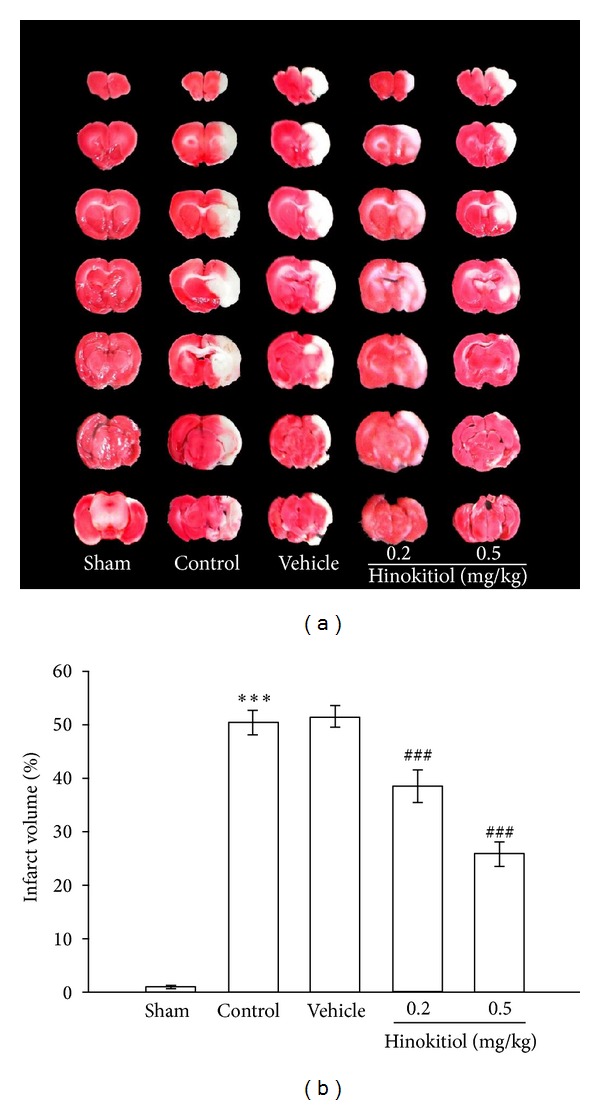
(a) Effects of hinokitiol in ischemia/reperfusion brain injury induced by MCAO in rats. Digital photographs show the infarct region in brain sections stained by 2% TTC 24 h after MCAO. (b) Dose-response effect of hinokitiol in ischemia/reperfusion brain injury induced by MCAO in rats. Rats were injected with solvent or hinokitiol (0.2 or 0.5 mg/kg, i.p.) at the time of 30 min before the onset of MCAO compared to sham control. Data are presented as percentage of contralateral hemisphere and expressed as means ± SEM. ***P < 0.001 compared with sham group; ^###^ *P* < 0.001 compared with vehicle control group (*n* = 10~15).

**Figure 3:**
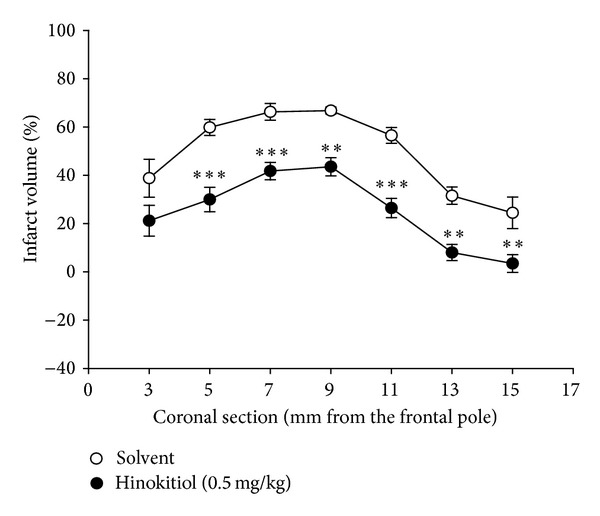
Effects of hinokitiol on the infarct volume of brain coronal section. Brains were dissected 24 h from reperfusion and sectioned at 2 mm thickness in the region from 1 mm to 15 mm of distance to the frontal pole. Data are expressed as means ± SEM. **P < 0.01 and ***P < 0.001 compared to the solvent-treated group.

The most commonly used tools for measuring the efficacy of putative neuroprotective compounds are TTC staining. In the present study, the cerebral infarction was examined using 2 mm-thick slices of the cerebrum 24 h after thromboembolic occlusion-induced reperfusion using 2% TTC staining. Typical photographs of the infarct region of TTC stained brain sections in embolic rats treated with hinokitiol are shown in Figure 2(a), where an intraperitoneal administration of hinokitiol at doses of 0.2 and 0.5 mg/kg significantly reduced infarct volume (white area) compared to the solvent-treated group in a dose-dependent manner (Figures 2(a) and 2(b)).  Figure 3 confers statistical results of the infarct areas of solvent- and hinokitiol- (0.5 mg/kg) treated groups at various distances from the frontal pole. The infarct area of the 3rd section was the largest in both groups than others. As shown in Figure 3, hinokitiol (0.5 mg/kg) decreased the area of infarction in the coronal section of MCAO-induced ischemic injury.

### 3.3. Hinokitiol Treatment Downregulates the Protein Expressions of HIF-1*α* and iNOS in Thromboembolic Cerebral Tissues

**Figure 4:**
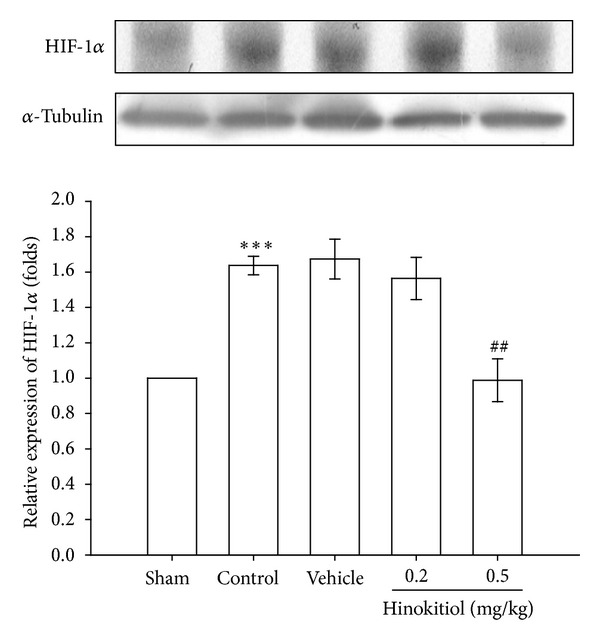
Effects of hinokitiol on the protein expression of HIF-1*α* in MCAO-reperfusion-induced cerebral homogenates. Rats were pretreated with hinokitiol (0.2 mg/kg or 0.5 mg/kg) before ischemia and compared with vehicle or sham control. All groups are represented as ipsilateral hemisphere. Data are expressed as the means ± SEM (*n* = 5). ***P < 0.001 compared with sham control group, ^###^ *P* < 0.001 compared with vehicle control group.

In order to investigate the effect of hinokitiol treatment on the inflammatory reaction in the ischemic brain, we measured the expression of HIF-1*α* and iNOS in thromboembolic occlusion-insulted cerebral tissues. As shown in Figure 4, HIF-1*α*, detected 24 h after thromboembolic occlusion-reperfusion injury, was more pronounced than the level obtained in the corresponding area of the sham-operated group. HIF-1*α* expression was significantly diminished by the treatment of hinokitiol at doses of 0.2 mg/kg (*P* < 0.05) and 0.5 mg/kg (*P* < 0.001) compared to the solvent-treated rats.

**Figure 5:**
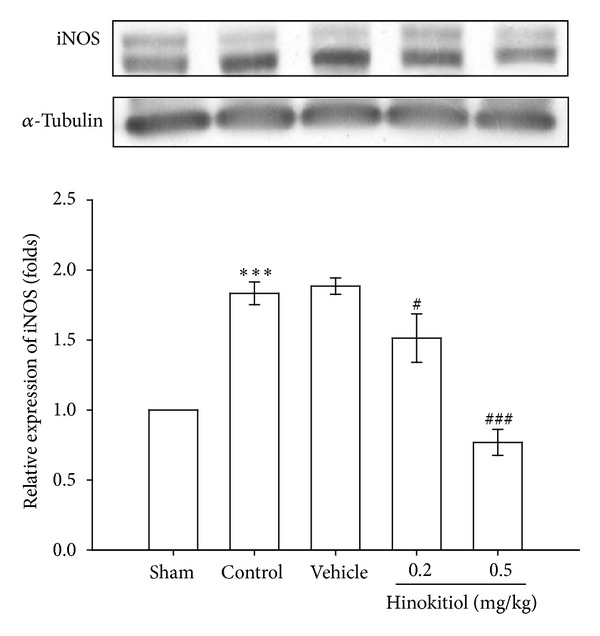
Effects of hinokitiol on the protein expression of iNOS in MCAO-reperfusion-induced cerebral homogenates. Rats were pretreated with hinokitiol (0.2 mg/kg or 0.5 mg/kg) before ischemia and compared with the vehicle group or the sham group. All groups are represented as ipsilateral hemisphere. Data are expressed as the means ± SEM (*n* = 5). ***P < 0.001 compared with sham control group; ^#^ *P* < 0.05 and ^###^ *P* < 0.001 compared with vehicle control group.

NO generated by the inducible form of NO synthase (iNOS) has been implicated in many pathophysiological states leading to myocardial dysfunction. In the present study, Figure 5 shows that the protein levels of iNOS in the brain of MCAO-injured group were higher compared to those in the sham groups. However, at doses of 0.2 mg/kg (*P* < 0.05) and 0.5 mg/kg (*P* < 0.001) hinokitiol treatment significantly inhibited iNOS protein expressions in the brain (Figure 5).

### 3.4. Hinokitiol Inhibits TNF-*α* Expressions in Thromboembolic Cerebral Tissues

**Figure 6:**
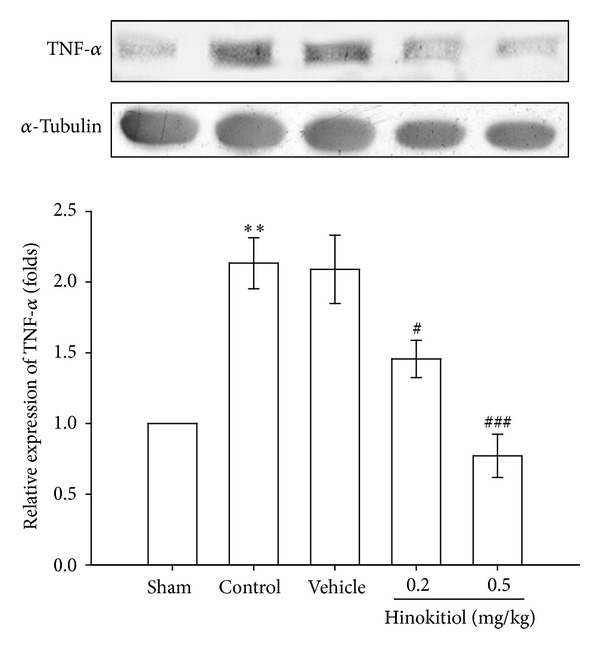
Effects of hinokitiol on the protein expressions of TNF-*α* in MCAO-reperfusion injury in rat cerebral homogenates. Rats were pretreated with hinokitiol (0.2 mg/kg or 0.5 mg/kg) before ischemia and compared with vehicle or sham control. All groups are represented as ipsilateral hemisphere. Data are expressed as the means ± SEM (*n* = 5). ***P < 0.01 compared with sham control group; ^#^ *P* < 0.05 and ^###^ *P* < 0.001 compared with vehicle control group.

Tumor necrosis factor-*α* (TNF-*α*) is one of the most typical proinflammatory cytokines with both beneficial and destructive properties of the central nervous system. Increasing lines of evidence have demonstrated that TNF-*α* plays an important role in the development of ischemic stroke. In the present study, MCAO-induced ischemia and reperfusion resulted in 1.23-fold elevation in the expression of TNF-*α* in the brain tissues. However, treatment with hinokitiol at doses of 0.2 and 0.5 mg/kg significantly decreased the expressions of TNF-*α* by *P* < 0.05 and *P* < 0.001, respectively (Figure 6).

### 3.5. Hinokitiol Treatment Downregulates the Expression of Active Caspase-3 in Thromboembolic Cerebral Tissues

**Figure 7:**
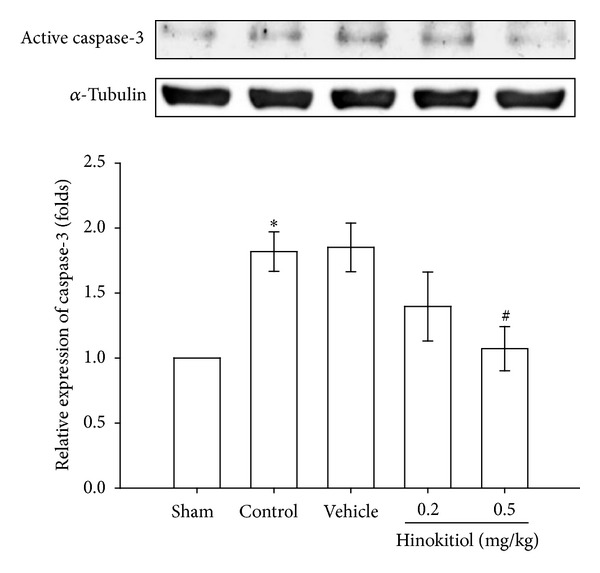
Effects of hinokitiol on the protein expression of caspase-3 in MCAO-induced rat ipsilateral brain hemisphere. Rats are pretreated with hinokitiol (0.2 mg/kg or 0.5 mg/kg) before ischemia and compared with vehicle or sham control. All groups are represented as ipsilateral hemisphere. Data are expressed as the means ± SEM (*n* = 5). *P < 0.05 compared with sham control group; ^#^ *P* < 0.001 compared with vehicle control group.

To further examine the molecular mechanisms underlying the neuroprotective effect of hinokitiol on thromboembolic occlusion-reperfusion, we investigated caspase-3 protein, which is implicated in apoptotic death. Caspase-3 is the most abundant cysteine protease in the brain and is acutely cleaved and activated in neurons in the early stages of reperfusion, leading to cell apoptosis. In this study, a significant increase in the expression of active caspase-3 was observed in the injured hemisphere of the transient thromboembolic occlusion-induced rats as compared to the level obtained in the corresponding area of the sham-operated group (Figure 7). Hinokitiol treatment at a dose of 0.5 mg/kg shows a significant inhibition (*P* < 0.05) of active caspase-3 expression in cerebral ischemic tissues (Figure 7). However, at a dose of 0.2 mg/kg, hinokitiol treatment was not effective on inhibiting the activation of caspase-3 in thromboembolic occlusion-induced rats.

## 4. Discussion

A central delayed mechanism beginning within hours from the onset of ischemia is the robust inflammatory response in the ischemic tissue [24]. There is increasing evidence showing a detrimental effect of the postischemic inflammatory reaction. Therefore, therapeutic strategies targeting the delayed inflammatory response could inhibit the progression of the tissue damage providing an extended therapeutic window for neuroprotection. Hinokitiol is a tropolone-related compound found in various natural sources such as the heartwood of several cupressaceous plants. Hinokitiol has been widely used in hair tonics, tooth pastes, cosmetics, and food as an antimicrobial agent [17]. It has also been shown to suppress tumor growth by inhibiting cell proliferation and inducing apoptosis in various carcinoma cell lines. Our recent study also demonstrates that hinokitiol has antiplatelet activity via inhibiting the PLCg2 and/or PKC cascades, and hydroxyl radical formation, followed by suppressing the activation of MAPKs and Akt [18]. In the present study, it is demonstrated, for the first time, that intraperitoneal administration of hinokitiol conceals thromboembolic stroke in rats by reducing the infarct volume, improves neurological outcome, and inhibits MCAO-reperfusion-induced expressions of TNF-*α*, HIF-1*α*, iNOS, and active caspase-3 protein expressions. Our recent study shows that hinokitiol exhibits antiplatelet activity *ex vivo* and anti-thrombogenic activity *in vivo* [18].

Hypoxia inducible factor-1 is a heterodimeric transcription factor that plays a pivotal role in regulating cellular O_2_ homeostasis. It is composed of an oxygen-regulated HIF-1*α* subunit and a constitutively expressed HIF-1*β* subunit. Under hypoxic conditions, hypoxia inhibits HIF-1*α* hydroxylation and allows its translocation to the nucleus, where it binds to HIF-1*β* to form an active complex HIF-1 and initiates the transcription of an array of target genes which are vital for cellular adaption to hypoxia [25]. Recently, it has been found that many nonhypoxic stimuli, such as cytokines, free radicals, growth factors, and hormones, can activate HIF-1*α* under normoxic conditions. Degradation HIF1-*α* is reported to be inhibited during hypoxia, allowing its rapid accumulation and binding to hypoxia responsive elements and thus activating the expression of hypoxia-responsive genes [26], many of which have a neuroprotective effect in the ischemic brain [27]. Because the iNOS gene contains the hypoxia-responsive enhancer (HRE) sequence to which HIF-1*α* binds [28], results from primary neuronal cultures of cells demonstrated that HIF-1*α* binds to the iNOS promoter gene under hypoxic conditions. Such binding is associated with an increase in iNOS expression [29]. Furthermore, HIF-1*α* combined with p53 may promote apoptotic cell death in ischemic areas [28].

A study was demonstrated that iNOS knock-out mice showed reduced brain damage after ischemia, because an increased expression of iNOS may also contribute to enhanced neuronal injury [30], and there is an evidence that iNOS plays a role as a mediator in the reduction of infarct size via late preconditioning [31]. A recent study also suggests that iNOS may be involved in the inflammatory reaction that follows cerebral ischemia, and iNOS mRNA and enzymatic activity are expressed in brain after permanent MCA occlusion [32]. Treatment with the selective iNOS inhibitor is reported to be reduced infarct volume, suggesting that iNOS activity contributes to ischemic brain damage [33]. In the present study, it has been demonstrated that the treatment of hinokitiol in MCAO-induced embolic rats significantly reduced the expression of HIF-1*α* and iNOS, harmful to the postischemic brain, and may be of worth in the treatment of cerebral ischemia.

Tumor necrosis factor-*α* (TNF-*α*) is one of the key immunomodulatory and proinflammatory cytokines upregulated during brain ischemia [34]. TNF-*α* was clinically correlated with acute hematoma enlargement, edema development, and poor patient outcome following spontaneous intracerebral hemorrhage (ICH) [35]. Similarly, increased TNF-*α* expression was observed in several different species and in multiple experimental models of ICH. Several studies have also observed a functional association between perihematomal TNF-*α* expression and the development brain edema and neurological injury after ICH [36, 37]. Moreover, based on experimental evidence, it has also been reported that higher circulating levels of TNF-*α* are associated with increased risks of stroke [38], and its administration during ischemic brain insult was shown to augment injury, as evidenced by increased tissue damage and neurological deficits [39]. Consistently, in this study, increased expression of TNF-*α* is directly correlated with neurological deterioration of brain tissues in MCAO-induced embolic rats. Therefore, agents with the ability to inhibit TNF-*α* expression are potentially beneficial in the treatment of stroke. An *in vitro* study has reported that hinokitiol obviously inhibited the production of LPS-induced TNF-*α* in activated macrophages in a dose-dependent manner without affecting cell viability [12]. These authors have also noticed that hinokitiol inhibits TNF-*α* more potently than other clinically available drugs such as pentoxifylline and theophylline. In this study, treatment of hinokitiol weakens the inflammatory response after embolic stroke in rats, as it is confirmed by the reduction of TNF-*α* expression in the ischemic brain.

Apoptosis occurs after transient cerebral ischemia and is regulated by the pro- and antiapoptotic proteins. Apoptosis contributes to ischemic cell damage after stroke [40]. Caspase-3 is normally an essential protein for brain development, but it also serves as a crucial mediator of neuronal apoptosis [41]. During ischemia, caspase-3 is cleaved and activated whereupon it degrades multiple substrates in the cytoplasm and nucleus leading to cell death [42]. Caspase-3-deficient adult mice reported to be more resistant to ischemic stress both *in vivo* and *in vitro* [42]. Therefore, it is of great interest to control the activation of caspase-3 for the potential therapeutic treatment of neurological diseases. Several studies have demonstrated that treatment of caspase-3 inhibitors reduced ischemic-induced brain damage [43]. Moreover, inhibition of caspase-3 like protease activity prevented DNA fragmentation in the ischemic brain [44]. In the present study, it shows that elevations of active caspase-3 occurred in MCAO-induced brain tissues was significantly suppressed by hinokitiol. This result suggests that hinokitiol plays a therapeutic role against embolic stroke by inhibiting caspase-3 expression.

In summary, hinokitiol treatment could improve the recovery of infarct volume and neurological outcome and provides neuroprotection in embolic stroke-induced ischemic rats. The inhibition of HIF-1*α* and TNF-*α*, followed by the inhibition of inflammatory responses (i.e., iNOS) and apoptosis (active caspase-3) by hinokitiol, may be the possible mechanisms involved to the observed neuroprotective effect of hinokitiol. Our findings provide evidence for the efficacy of hinokitiol as a neuroprotective agent, with a stupendous therapeutic window for the prevention of ischemic brain injury.
